# IgA Nephropathy in Salvador, Brazil. Clinical and laboratory presentation at diagnosis

**DOI:** 10.1590/2175-8239-JBN-3851

**Published:** 2018-05-07

**Authors:** Brenda Navarro de Souza, Maria Brandão Tavares, Maria Fernanda Sanches Soares, Washington Luis Conrado dos Santos

**Affiliations:** 1 Fundação Oswaldo Cruz, Brasil, Instituto Gonçalo Moniz, Salvador, BA, Brasil.; 2 Escola Bahiana de Medicina e Saúde Pública, Salvador, BA, Brasil.; 3 John Radcliffe Hospital, OUH NHS Foundation Trust, USA.

**Keywords:** Glomerulonephritis, Glomerulopathy, IgA, Classification, Glomerulonefrite, Glomerulopatia, IgA, Classificação

## Abstract

**Introduction:**

IgA nephropathy (IgAN) is the most prevalent primary glomerulopathy in the world, but great variation is reported in different countries. In Brazil, the reported prevalence is high in the Southeastern States and low in Salvador, Bahia State, Brazil.

**Objectives:**

This study investigated the clinical and histological patterns of patients with IgAN in Salvador, Brazil.

**Methods:**

This is a descriptive study that included all patients with a diagnosis of IgAN performed in native kidney biopsies collected from referral nephrology services of public hospitals in Salvador between 2010 and 2015. Results: Thirty-two cases of IgAN were identified, corresponding to 6% of primary glomerulopathies. There was a slight male predominance (56%) and the median age was 30 [22-40] years. Hematuria was present in 79%, non-nephrotic proteinuria was present in 61%, and hypertension was present in 69% of patients. Segmental sclerosis (S1 lesions) was present in 81% of cases, and chronic tubulo-interstitial lesions (T1 and T2 lesions) were present in 44% of cases. Patients with M1 and T2 MEST-C scores exhibited higher serum urea and creatinine than other patients.

**Conclusion:**

The prevalence of IgAN was lower in Salvador than other regions of Brazil. Chronic histological lesions and laboratory markers of severe disease were frequent. M1 and T2 MEST-C scores were correlated with markers of renal dysfunction.

## RESUMO

**Introdução:**

A nefropatia por IgA (NIgA) é a glomerulopatia primária mais prevalente no mundo, mas grande variação é relatada em diferentes países. No Brasil, a prevalência relatada é alta nos estados do Sudeste e baixa em Salvador, Bahia, Brasil.

**Objetivos:**

Este estudo investigou os padrões clínicos e histológicos de pacientes com NIgA em Salvador, Brasil.

**Métodos:**

Trata-se de um estudo descritivo que incluiu todos os pacientes com diagnóstico de NIgA, realizados em biópsias de rins nativos, coletados nos serviços de referência em nefrologia dos hospitais públicos de Salvador, entre 2010 e 2015. Resultados: Foram identificados 32 casos de NIgA, correspondendo a 6% de glomerulopatias primárias. Houve uma ligeira predominância do sexo masculino (56%) e a mediana da idade foi de 30 [22-40] anos. Hematúria esteve presente em 79%, proteinúria não nefrótica esteve presente em 61% e hipertensão esteve presente em 69% dos pacientes. A esclerose segmentar (lesão S1) estava presente em 81% dos casos, e lesões túbulo-intersticiais crônicas (lesões T1 e T2) estavam presentes em 44% dos casos. Pacientes com escores M1 e T2 MEST-C exibiram maior ureia e creatinina séricas que outros pacientes.

**Conclusão:**

A prevalência de NIgA foi menor em Salvador do que em outras regiões do Brasil. Lesões histológicas crônicas e marcadores laboratoriais de doença grave foram frequentes. Os escores M1 e T2 MEST-C foram correlacionados com marcadores de disfunção renal.

## INTRODUCTION

IgA nephropathy (IgAN) is the most prevalent glomerular disease worldwide.^1^^,^ 2 However, the estimated prevalence of IgAN in biopsy samples displays wide variation in different countries and in different regions of the same country, such as Brazil.^3^^,^ 4 ^,^ 5 Differences in the frequency of IgAN are attributed to ethnical background or selection bias, which stems from heterogeneous biopsy indication policies.^2^ For example, the prevalence of IgAN is high in Japan (30%), Italy (35%), and Spain (15%)*,* and it is becoming the most prevalent glomerular disease in these areas.^6^^,^ 7 ^,^ 8 However, it is much lower in Saudi Arabia (4.7%), Africa (2.8%), India (8.1%), Colombia (11.8%), Peru (1.5%), and Mexico (7%).^9^^,^ 10 ^,^ 11 ^,^ 12 ^,^ 13 ^,^ 14 The prevalence of IgAN in the USA is high in Caucasian (38%) and East Asian populations (36%), and low in African-American (3%) and Hispanic populations (19%).^15^ The estimated prevalence of IgAN varies in different Brazilian States. It is high in the Southeastern States of Minas Gerais (16.15%) and São Paulo (17.8%), and it is low in the States of Para (6.3%), Amazonas (4.3%), and Bahia (5%).^16^^,^ 17 ^,^ 18 ^,^ 4 ^,^ 19

The estimated prevalence of IgAN in Salvador, BA, Brazil is 7% of primary glomerular diseases (dos-Santos *et al*., in press).[1] Salvador’s ethnical background might be accountable for this low figure: approximately 73% of the population self-declares as being of African heritage according to the Brazilian Institute of Geography and Statistics (IBGE). Comparatively, the estimated population self-declared of African heritage is 27.2% and 45.4% in São Paulo and Minas Gerais (IBGE), respectively, where the prevalence of IgAN are 17.8% and 16.15%, respectively.^16^^,^ 17 Conversely, the estimated self-declared African heritage in the State of Para is 71.9% (IBGE), whereas the prevalence of IgAN is 6.3%.^18^ However, it is not clear whether these differences are due to the ethnic backgrounds or to different criteria to indicate kidney biopsy.

The present study investigated the pattern of clinical and histological presentations of patients with IgAN from Salvador, Brazil, at disease diagnosis. We aimed to shed light on the actual disease prevalence in this highly African-decent populated area in Northeastern Brazil.

## METHODS

*Cases:* This report is a descriptive exploratory study of all biopsy-proven IgAN cases diagnosed in referral nephrology services of public hospitals in Salvador, State of Bahia, Brazil, and examined at the Gonçalo Moniz Institute, Fiocruz (IGM-Fiocruz) between 2010 and 2015. Only native kidney biopsies with available and sufficient histologic material and clinical records were included.

*Renal biopsies:* All renal biopsies underwent: 1) routine light microscopy processing (fixed in Bouin’s solution, embedded in paraffin, sectioned at 2-µm thickness, and stained with hematoxylin and eosin, Periodic Acid Schiff, Periodic Schiff-Methenamine Silver, Azan, and Picro Sirius red); and 2) immunofluorescence processing (embedded in cryopreservation medium and incubated with antisera anti- IgA, IgG, IgM, kappa chains, lambda chains, C1q, C3, and fibrinogen). All samples were fixed in 1% glutaraldehyde in cacodylate buffer, post-fixed in osmium tetroxide, and embedded in Poly/Bed^®^ for ultrastructural analysis when required.

*Histological analysis*: Two pathologists (MFSS and WLCS) without previous knowledge of the reported pattern of renal lesion independently reviewed the histological slides of each patient. Discrepancies in independent analyses between the two pathologists were resolved in a consensus analysis. The histological analyses are classified according to the Oxford classification of IgAN (MEST-C scores).^20^^,^ 21

*Clinical data:* The following data were obtained from the biopsy request forms: Age, sex, presence of systemic arterial hypertension, nephrotic syndrome, presence and amount of proteinuria, presence of macroscopic and microscopic hematuria, markers of renal function (serum urea and creatinine), serum albumin, total cholesterol, triglycerides, biopsy date, and registry of the material received for examination (light microscopy). The upper age threshold for pediatric cases was set as less than or equal to 16 years. Nephrotic syndrome and the presence of proteinuria and macroscopic hematuria were considered when listed in the biopsy request form. Nephrotic proteinuria was considered when > 3.5 g/24 h, or its presence was described in the biopsy request form. Microscopic hematuria was considered when the presence of more than five red cells per field was reported in the urine summary or described on the biopsy request form.

*Data analysis:* Data are reported as percentages and absolute numbers, and summarized as means ± standard deviations or medians and the 25% and 75% percentiles. Data were summarized using Prism 5.01 (GraphPad, San Diego, CA, USA) and StataIC11 software.

*Ethical considerations:* The study was performed in accordance with resolution No. 196/96 of the National Health Council, and the Ethics Committee for Research Involving Human Subjects of the Instituto Gonçalo Moniz; Fiocruz approved the procedure (Protocol No. 1642146).

## RESULTS

*General patient characteristics:* A total of 1,045 renal biopsies were examined in the IGM-Fiocruz between 2010 and 2015. However, 134 biopsies were from transplanted kidneys, 110 had underrepresented renal parenchyma (mostly due to the absence of glomerulus for immunofluorescence), seven cases had an inconclusive diagnosis, and two cases were received for a second opinion. A total of 253 cases were excluded from the study, and 792 cases were included. Of the included cases, 556 were primary glomerulopathy and 236 were secondary glomerulopathy. Thirty-two cases were diagnosed as IgAN, and one case presented clinical findings suggestive of Henoch-Shoenlein vasculitis. Therefore, the prevalence of IgAN was 6% in the primary glomerulopathy cases and 4% in the renal biopsies of native kidneys.

Table 1 shows the primary clinical and demographic characteristics of these patients. Age varied from 2 to 59 years with a median of 30 (22-40; first and third quartiles, respectively) years. Four (12.5%) patients were children, and 28 (87.5%) patients were adults, with a slight male predominance.

**Table 1:** General characteristics of patients with IgA nephropathy who underwent renal biopsy in Salvador, BA, Brazil between 2010 and 2015

PARAMETER (*N*)	VALUE	(%) [Q1-Q3]
Patients	32	(100%)
Sex: (32)		
Female	14	(44%)
Male	18	(56%)
Age		
Median	30	[22-40]
Range	2-	59
Clinical presentation:		
Hematuria (28)	22	(79%)
Microscopic	18	(64%)
Macroscopic	13	(46%)
Systemic arterial hypertension (26)	18	(69%)
Non-nephrotic Proteinuria (28)	17	(61%)
Nephrotic syndrome (28)	11	(39%)
Laboratory tests:		
Proteinuria (g/24 h) (19)	2.0	[1.3-4.0]
Serum albumin (g/dL) (31)	3.1	[3.0-4.0]
Serum urea (mg/dL) (31)	41	[28-74]
Serum creatinine (mg/dL) (31)	1.1	[0.9-2.5]
Serum cholesterol (mg/dL) (16)	214	[179-288]
Serum triglycerides (mg/dL) (16)	208	[109-344]

The primary reported clinical presentations were hematuria in 22/28 (79%), systemic hypertension in 18/26 (69%), and non-nephrotic proteinuria in 17/28 (61%) of cases.

**Figure 1:**
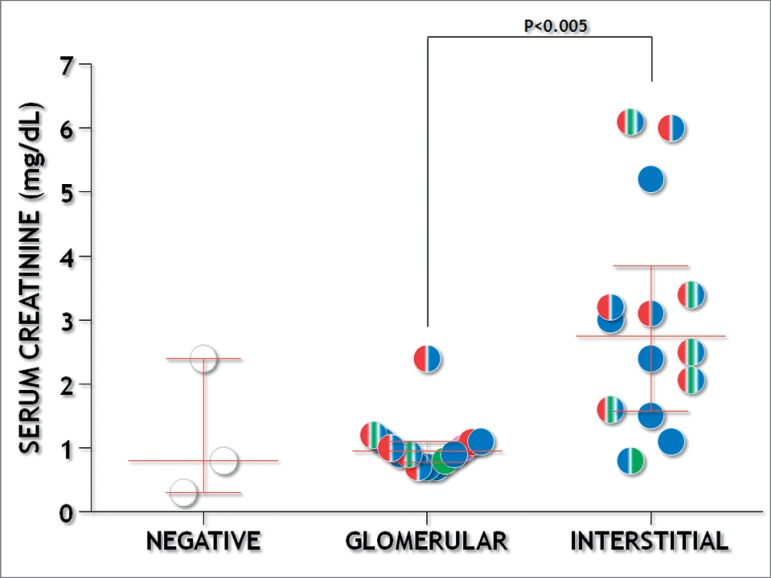
Glomerular and tubule-interstitial MEST-C scores and serum creatinine concentrations. Colors represent positive MEST-C scores: M1-red, E1-green, S1-blue.

**Figure 2:**
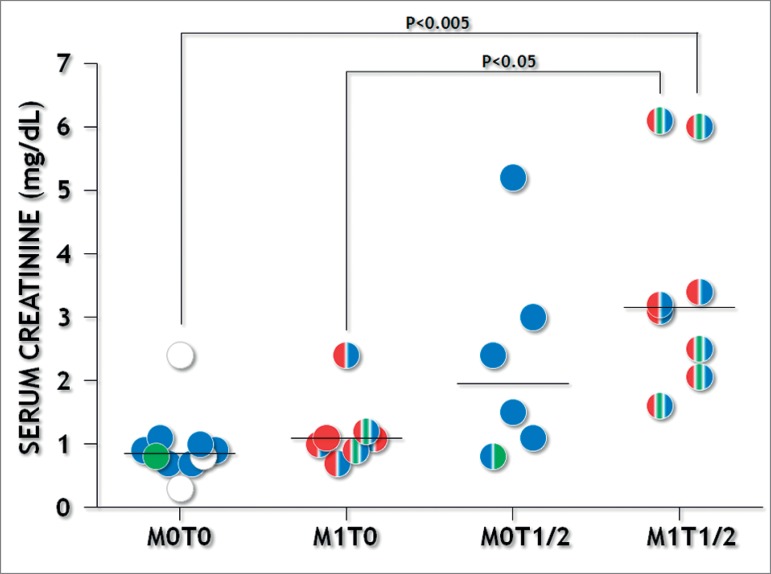
M1 and T1/2 scores combination and serum creatinine concentrations. Colors represent positive MEST-C scores: M1-red, E1-green, S1-blue.

Table 2 shows the distribution of MEST-C scores of the renal biopsies of the IgAN patients. The most common lesion observed was segmental sclerosis (26; 81%). There was a predominance of chronic sclerosis over proliferative glomerular lesions. As expected, chronic tubule-interstitial lesions were associated with renal dysfunction (Fig. 1). Serum urea and creatinine concentrations were higher in patients with the combination of M1 and T2 scores (Table 2 and Fig. 2). There was a trend for increased proteinuria, serum urea, and creatinine concentrations in individuals with S1 compared with patients with S0, but this difference was not statistically significant. Furthermore, all the six patients with S0 also had T0, while 14/26 (54%) patients with S1 had T1 or T2. Such association was statistically significant (Fisher’s test *p* = 0.02).

**Table 2:** MEST-C scores and distribution of laboratory variables in patients with IgAN subjected to kidney biopsy in Salvador, Brazil, 2010-2015

MEST	N		UREA		CREATININE		ALBLBUMIN		CHOLESTEROL		TRIGLYCERIDES		24 h PTU	
ALL	32	(100)	41	[28-74]	1.1	[0.9-2.5]	3.1	[3.0-4.0]	213	[179-288]	208	[109-344]	2.0	[1.3-4.0]
M0	17	(53)	30	[22-59]^a^	1.0	[0.8-1.9]^b^	3.1	[2.5-4.0]	220	[198-368]	103	[71-354]	1.9	[1.2-2.0]
M1	15	(47)	67	[36-85]^a^	2.1	[1.1-3.2]^b^	3.1	[3.0-4.0]	187	[172-259]	212	[201-335]	2.3	[2.2-4.7]
E0	23	(72)	37	[25-74]	1.1	[0.9-2.4]	3.1	[3.0-4.0]	211	[185-318]	214	[86-318]	1.7	[1.2-3.1]
E1	9	(28)	42	[36-83]	1.6	[0.9-2.5]	3.6	[3.0-4.0]	216	[173-269]	202	[125-350]	2.3	[2.0-5.1]
S0	6	(19)	28	[21-30]	0.8	[0.8-1.1]	2.0	[1.5-4.1]	417	[216-513]	354	[103-578]	1.2	[0.4-2.0]
S1	26	(81)	46	[31-74]	1.4	[0.9-3.0]	3.1	[3.0-4.0]	189	[173-249]	204	[115-283]	2.2	[1.5-4.0]
T0	18	(56)	30	[23-36]^c^ ^,^ ^d^	0.9	[0.8-1.1]^e^	3.5	[2.4-4.0]	216	[189-308]	216	[100-354]	1.5	[1.2-2.2]
T1	8	(25)	60	[46-88]^c^	2.0	[1.3-2.8]	3.1	[2.8-3.6]	173	[170-225]	204	[201-514]	3.0	[1.9-5.1]
T2	6	(19)	74	[65-130]^d^	4.2	[3.1-6.0]^e^	3.1	[3.0-4.0]	227	[169-294]	174	[125-248]	2.2	[2.1-3.5]
C0	25	(78)	39	[79-28]	1.1	[2.8-0.8]	4.0	[4.0-3.0]	189	[249-173]	208	[335-103]	2.0	[2.8-1.3]
C1	7	(22)	41	[51-25]	1.2	[2.1-0.9]	3.1	[2.1-2.5]	269	[308-225]	250	[364-135]	3.4	[4.7-2.2]

Mann-Whitney or Kruskall-Wallis tests were applied where applicable.

a, b and c *p* < 0.05;

d and e *p* < 0.005. (%), [Q1-Q3].

## DISCUSSION

This study is the first report on the characteristics of patients with IgAN in Salvador, Brazil. This glomerular disease is considered rare in Bahia State because it has not been reported in most of the previous studies on glomerular diseases in this part of the country.^22^^,^ 23 A recent survey between 2003 and 2010 demonstrated a prevalence of 5% of kidney diseases and 7% of primary glomerular diseases (dos-Santos *et al*., in press),[1] which is similar to the results of this study, as well as to the rates reported in non-Caucasian and non-Asiatic populations. However, it is lower than the rates reported in other parts of Brazil. This low prevalence of IgAN may be explained by the ethnical constitution of Salvador’s population, which is largely Afro-descendant. Another potential explanation may be the criteria for renal biopsy indication used by assistant nephrologists, who favor kidney biopsy in patients with nephrotic syndrome, keeping with the standard of practice in Brazil.^24^ The clinical presentation of most patients in this study was hematuria and minor urinary changes, which shows that these conditions are also relevant for kidney biopsy indication in referral nephrology centers in Salvador. However, the proportion of macroscopic hematuria and nephrotic proteinuria reported in this work is among the highest reported in the literature, which suggests that cases of microscopic hematuria, either isolated or combined with other minor urinary changes, may be overlooked.^25^^,^ 26 Further studies are necessary to exclude a potential selection bias for biopsy indication.

The demographic characteristics and clinical presentation of patients in this work such as age, sex, frequency of hematuria, and non-nephrotic proteinuria, are similar to previously published studies.^20^^,^ 27 ^-^ 29 Microscopic hematuria with minimal proteinuria is the most common presentation of IgAN, and it is associated with a favorable prognosis. In contrast, the presence of significant proteinuria, hypertension, and decreased glomerular filtration rate is related to a poor prognosis. The median protein concentration in urine was slightly higher in this study than previously published studies,^20^^,^ 25 ^,^ 28 and hypertension was recorded in 69% of patients. These data suggest that patients with IgAN reported here had already evolved to a late stage of progression to chronic kidney disease at the time of biopsy.

Our series demonstrated a high frequency of positive MEST-C scores for segmental sclerosis (81%) and tubule-interstitial lesion (44%). Other authors reported similar proportions.^26^^,^ 28 Together, positive T or M scores were associated with increased serum urea and creatinine concentrations in the present study. Tubulo-interstitial changes are consistently associated with the clinical presentation and outcome of IgAN.^28^^,^ 29 Despite associations between M1 and renal dysfunction being less well established, Lee *et al*. demonstrated an M1 association with disease progression.^25^^,^ 28 A model for using MEST scores for renal outcome at the time of biopsy has been proposed by Barbour and colleagues (2016).^30^ The authors propose that a combination of MEST score with the data on blood pressure, proteinuria, and eGFR at the time of biopsy may predict the renal outcome similar to using clinical data over 2 years of follow-up. Further development of models of association between combined MEST-C scores and clinical presentation or outcome of IgAN are still required.

The observation of a high proportion of positive MEST-C scores for segmental sclerosis and tubule-interstitial lesions in this study combined with the severity of clinical presentation indicates that patients with IgAN in Salvador are in an advanced stage of the disease when subjected to renal biopsy. Further studies will be useful to determine factors associated with the severity of IgAN presentation and prognosis in this city.

## CONCLUSION

1. The prevalence of IgAN in patients submitted to renal biopsies in Salvador, Bahia is among the lowest reported in Brazil.
2. Patients with IgAN in this series presented high protein concentrations in urine and a high frequency of hypertension, which suggests a late stage of CKD progression.
3. A high frequency of positive MEST scores associated with progressive kidney disease was observed in these patients: segmental sclerosis (81%) and tubule-interstitial lesion (44%).
4. Positive T or M scores were associated with increased serum urea and creatinine concentrations.

## LIST OF ABBREVIATIONS

IgAN - IgA nephropathy. CKD - Chronic kidney disease. IBGE - Brazilian Institute of Geography and Statistics.
